# Toxic effects of selected proprietary dry eye drops on *Acanthamoeba*

**DOI:** 10.1038/s41598-018-26914-3

**Published:** 2018-06-04

**Authors:** Ines Sifaoui, María Reyes-Batlle, Atteneri López-Arencibia, Olfa Chiboub, Javier Rodríguez-Martín, Pedro Rocha-Cabrera, Basilio Valladares, José E. Piñero, Jacob Lorenzo-Morales

**Affiliations:** 1 Laboratoire Matériaux-Molécules et Applications, IPEST, B.P 51 2070, University of Carthage, La Marsa, Tunisia; 2 University Institute of Tropical Diseases and Public Health, University of La Laguna, Avda Francisco Sanchez s/n, Campus de Anchieta, 38271, La Laguna, Santa Cruz de Tenerife, Canary Islands, Spain; 3 Clínica Nivaria, Santa Cruz de Tenerife, Canary Islands, Spain; 4 Department of Ophthalmology, Hospital Universitario de Canarias, Santa Cruz de Tenerife, Canary Islands, Spain

## Abstract

Amoebae of the genus *Acanthamoeba* are ubiquitous protists that have been isolated from many sources such as soils, water and the air. They are responsible for infections including fatal encephalitis and a severe keratitis in humans. To date, there is no satisfactorily effective therapeutic agent against this pathogen and the infections it causes are exacerbated by the existence of a resistant cyst stage produced by this amoeba. As dry eye syndrome is a risk factor for *Acanthamoeba* keratitis, we aimed to evaluate the anti-*Acanthamoeba* activity of a variety of proprietary eye drops intended to treat dry eye syndrome. From the nine eye drop formulations tested, “Systane Ultra” was determined to be the most active against all tested *Acanthamoeba* strains. During our investigations into the mode of action of Systane Ultra, we discovered that it decreases mitochondrial membrane potential and ATP levels, induces chromatin condensation, and increases the permeability of the plasma-membrane.

## Introduction

*Acanthamoeba* keratitis (AK) is increasingly being recognized as a serious infection of the cornea that can lead to a permanent visual impairment or even blindness^1^. In the developed world, AK is most often found in contact lens users particularly where poor hygiene has been practiced. AK is difficult to diagnose partly because clinicians rarely encounter this infection but also because the symptoms mimic those of other types of keratitis diseases (viral, bacterial and fungal). Patients with AK may experience eye pain and redness, blurred vision, photophobia and excessive tear production^1^. Dry eyes disease (DED) is a more common ocular surface disease that has a severe impact both on quality of live and on cost but it is also a predisposing risk factor for the development of AK^2^. DED results from either a systemic immunologic disorder known as Sjögren’s syndrome^3^ in which there is insufficient production of moisture in the salivary and tear-producing glands, or from the low production or high evaporation of tears caused by other means^4^. Its severity may range from mild/episodic to severe/chronic and the disease is characterized by several symptoms including visual disturbance (blurred and fluctuating vision), foreign-body sensation and eye discomfort, irritation, ocular surface inflammation, redness, excess tearing, and photophobia^5^. DED is treated with a range of proprietary eye drops which contain a variety of active ingredients. We could find no previous studies describing the potential anti-*Acanthamoeba* activity of eye dry drops, and so the aim of the present study was to assess the potential anti-amoebic activity of several eye dry drops solutions, against a range of *Acanthamoeba* strains.

## Material and Methods

### Chemicals

Nine proprietary eye drop solutions available commercially for topical use against DED were selected for analysis. Table 1 shows the details of the composition of these solutions.

**Table 1:** Eye dry drops Composition.

Eye drop solution	Composition
Optiben	0.2% Sodium hyaluronate without preservative agents
Thealoz Duo	3% Trehalose 0.15% Sodium hyaluronate
Colircusi Humectante	0.3% Hypromellose 0.55% Sodium chloride
Systane Ultra	0.4% Polyethylene Glycol 400 0.3% Propylene Glycol
Optava Fusion	0.5% Carmellose sodium
Artelac splash	0.2% Sodium hyaluronate without preservative agents
Monoprost	50 µg/ml of latanoprost
Visionlux	0.3% Sodium hyaluronate without preservative agents
Relive Total Care	1.5% - Polyvinylpyrrolidone K30 (PVP K30)/Actinoquinol/Cyanocobalamin

### *In vitro* drug sensitivity assay

#### Strains used

The anti-*Acanthamoeba* activity of the selected eye drops were initially evaluated against the *Acanthamoeba castellanii* Neff (ATCC 30010) type strain from the American Type Culture Collection. Subsequently, eye drop solutions were tested against three clinical isolates, CLC-16 and *Acanthamoeba griffini*, genotype T3 and CLC-51, genotype T1 obtained in previous studies^6,7^. Those strains were grown axenically in PYG medium (0.75% (w/v) proteose peptone, 0.75% (w/v) yeast extract and 1.5% (w/v) glucose) containing 40 μg gentamicin ml^−1^ (Biochrom AG, Cultek, Granollers, Barcelona, Spain).

### *In vitro* effect against the trophozoite stage of ***Acanthamoeba***

The anti-*Acanthamoeba* activities of the eye drop solutions were determined by the Alamar Blue assay as previously described^6,8^. Briefly, *Acanthamoeba* strains were seeded in duplicate on a 96-well microtiter plate with 50 μl from a stock solution of 10^4^ cells ml^−1^. Amoebae were allowed to adhere for 15 min and 50 μl of serial dilution series of the eye drop solution was added. Finally, the Alamar Blue Assay Reagent (Bioresource, Europe, Nivelles, Belgium) was added into each well at an amount equal to 10% of the medium volume. The plates were then incubated for 120 h at 28 °C with a slight agitation. Subsequently the plates were analyzed, during an interval of time between 72 and 144 h, with an Enspire microplate reader (PerkinElmer, Massachusetts, USA) using a test wavelength of 570 nm and a reference wavelength of 630 nm. Percentages of growth inhibition, 50% and 90% inhibitory concentrations (IC_50_ and IC_90_) were calculated by linear regression analysis with 95% confidence limits. All experiments were performed three times each in duplicate, and the mean values were calculated.

### *In vitro* effect against the cyst stage of *Acanthamoeba*

The cysticidal activity was determined by the Alamar Blue assay at 144 h and confirmed visually by inverted microscopy. *A*. *castellanii* Neff cysts were prepared as described by Lorenzo-Morales *et al*.^9^. Briefly, trophozoite were transferred from PYG medium based cultures (trophozoite medium) to Neff’s encystment medium (NEM; 0.1 M KCl, 8 mM MgSO_4_·7H_2_O, 0.4 mM CaCl_2_·2H_2_O, 1 mM NaHCO_3_, 20 mM ammediol [2-amino-2-methyl-1,3-propanediol; Sigma Aldrich Chemistry Ltd., Madrid, Spain], pH 8.8, at 25 °C) and were cultured in this medium with gently shaking for a week in order to obtain mature cysts. After that, mature cysts were harvested and washed twice using PYG medium.

A serial dilution of the eye drops was made in PYG. The *in vitro* susceptibility assay was performed in sterile 96-well microtiter plates (Corning™). To these wells the drug concentration to be tested and 5*10^4^ mature cysts of *Acanthamoeba*/ml were added. The final volume was 100 μL in each well. Finally, 10 μL of the Alamar Blue Assay Reagent (Biosource, Europe, Nivelles, Belgium) was placed into each well, and the plates were then incubated for 144 h at 28 °C with slight agitation. Subsequently the plates were analyzed, with an Enspire microplate reader (PerkinElmer, Massachusetts, USA) using a test wavelength of 570 nm and a reference wavelength of 630 nm. Percentages of growth inhibition, 50% and 90% inhibitory concentrations (IC_50_ and IC_90_) were calculated by linear regression analysis with 95% confidence limits. All experiments were performed three times each in duplicate, and the mean values were calculated.

### Double-stain assay for programmed cell death determination

A double-stain apoptosis detection kit (Hoechst 33342/PI) (GenScript, Piscataway, NJ, USA) and an inverted confocal microscope (Leica DMI 4000B) were used. The experiment was carried out by following the manufacturer’s recommendations, and 10^5^ cells/well were incubated in a 24-well plate for 24 h with the previously calculated IC_50_ and IC_90_. The double-staining pattern allows the identification of three groups in a cellular population: live cells will show only a low level of fluorescence, cells undergoing PCD will show a higher level of blue fluorescence (as chromatin condenses), and dead cells will show low-blue and high-red fluorescence (as the propidium Iodide stain enters the nucleus).

### Plasma membrane permeability

The SYTOX Green assay was performed to detect the parasite’s membrane permeability alterations. Briefly, 10^5^ trophozoite were washed and incubated in saline solution with the SYTOX Green at a final concentration of 1 μM (Molecular Probes) for 15 min in the dark. Subsequently the test eye drop solution was added (IC_90_). After 24 h of treatment, cells were observed in a Leica TSC SPE- confocal microscope equipped with inverted optics at λexc = 482 nm and λem = 519 nm^9,10^.

### Analysis of Mitochondrial Membrane Potential

The collapse of an electrochemical gradient across the mitochondrial membrane during apoptosis was measured using a JC-1 mitochondrial membrane potential detection kit (Cell Technology) by flow cytometry as described by the manufacturer. After being treated with IC_50_ and IC_90_ of the test solution for 24 h, the cells were centrifuged (1000 r.p.m. × 10 min) and resuspended in JC-1 buffer. 100 µl of each treated culture was added to a black 96 well plate than 10 µl of JC-1 was added and incubated at 26 °C for 30 min. The mean green and red fluorescence intensity was measured using flow cytometry for 30 minutes.

### Measurement of ATP

ATP level was measured using a CellTiter-Glo Luminescent Cell Viability Assay. The effect of the drug on the ATP production was evaluated by incubating (10^5^) of cells/ml with the previously calculated IC_50_ and IC_90_ of the active eye drop solution.

## Results

### *In vitro* drug sensitivity assay

Initially, all eye drops were screened for their activity against the trophozoite stage of *Acanthamoeba castellanii* Neff strain. The IC_50_ and IC_90 at_/96 h were chosen as the appropriate and comparable data to give as previously described^6^. The results are illustrated in Table 2.

**Table 2:** Activity against *Acanthamoeba castellanii* Neff.

Eye drop solution	IC_50_(%)	IC_90_(%)
Optiben	3.796 ± 0.294	26.384 ± 2.119
Thealoz Duo	47.941 ± 4.069	—
Colircusi Humectante	7.410 ± 0.496	36.300 ± 4.091
Systane Ultra	2.036 ± 0.137	14.505 ± 2.414
Optava Fusion	13.763 ± 2.610	>50
Artelac Splash	47.946 ± 3.770	>50
Monoprost	NA	NA
Visionlux	11.488 ± 0.398	>50
Relive Total Care	NA	NA

Among the nine tested eye drops, seven of them are active against trophozoites with an IC_50_ ranged from 2.036 ± 0.137% (v:v) for Systane Ultra to 47.946 ± 3.770% (v:v) for Artelac Splash. Based on their amoebicidal activity on the Neff strain, three eye drop solutions, namely Systane Ultra, Colircusi Humectante and Optiben were selected to evaluate their effect on the clinical *Acanthamoeba* strains. The results are illustrated in Table 3.

**Table 3:** Activity of Colircusi Humectante, Systane Ultra and Optiben against different strains of *Acanthamoeba* IC_50_ (%) and IC_90_ (%).

		Systane Ultra	Colircusi Humectante	Optiben
*Acanthamoeba* *castellanii* Neff	IC_50_	2.036 ± 0.137	7.410 ± 0.496	3.796 ± 0.294
	IC_90_	14.505 ± 2.414	36.300 ± 4.091	26.384 ± 2.119
*Acanthamoeba griffini*	IC_50_	10.691 ± 1.484	15.743 ± 2.893	29.325 ± 1.093
	IC_90_	26.584 ± 0.622	32.057 ± 3.303	>50
CLC51	IC_50_	3.402 ± 0.183	4.313 ± 0.030	4.642 ± 0.280
	IC_90_	8.039 ± 0.254	7.897 ± 0.134	8.029 ± 0.176
CLC 16	IC_50_	5.762 ± 0.340	5.367 ± 0.435	5.846 ± 0.527
	IC_90_	11.363 ± 1.912	9.509 ± 0.950	9.639 ± 0.716

**Figure 1:**
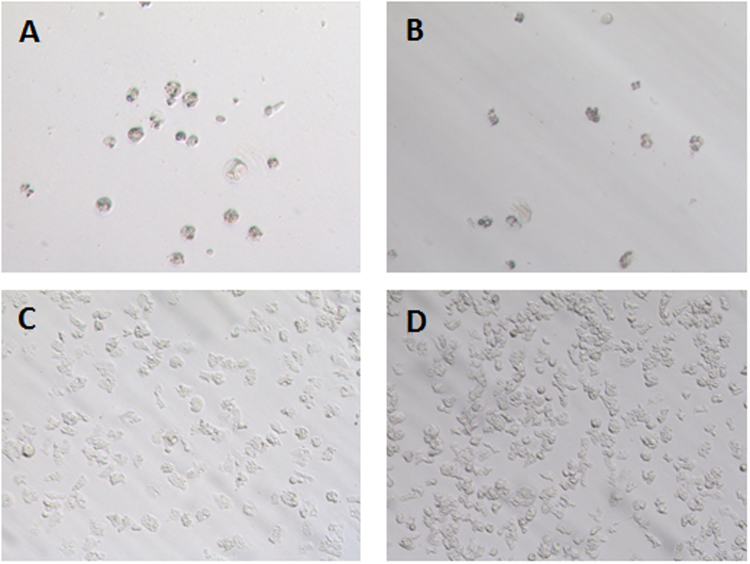
Effect of Systane Ultra at 12.5% against *Acanthamoeba castellanii* Neff (**A**) and and *A*. *griffini* strain (**B**) trophozoites observed by inverted microscopy (x20). Negative control (untreated strains) *Acanthamoeba castellanii*. Neff (**C**) and *A*. *griffini* (**D**), at 96 hours of incubation respectively.

The analysis of variance by Multifactor ANOVA, illustrated that the biological activity was strain dependent with *p* = 0.0001 < 0.001. In fact, the *Acanthamoeba castellanii* Neff was the most sensitive strain to the eye drops. Meanwhile, *A*. *griffini* was the most resistant strain to all eye drops. The toxic effect was statistically significant with *p* = 0.0000 < 0.001, Systane Ultra was statistically the most effective drug against all the strains with the IC_50_ ranged from 2.036 ± 0.137% for the *A*. *castellanii* Neff to 10.691 ± 1.484% for *A*. *griffini* (Fig. 1).

**Figure 2:**
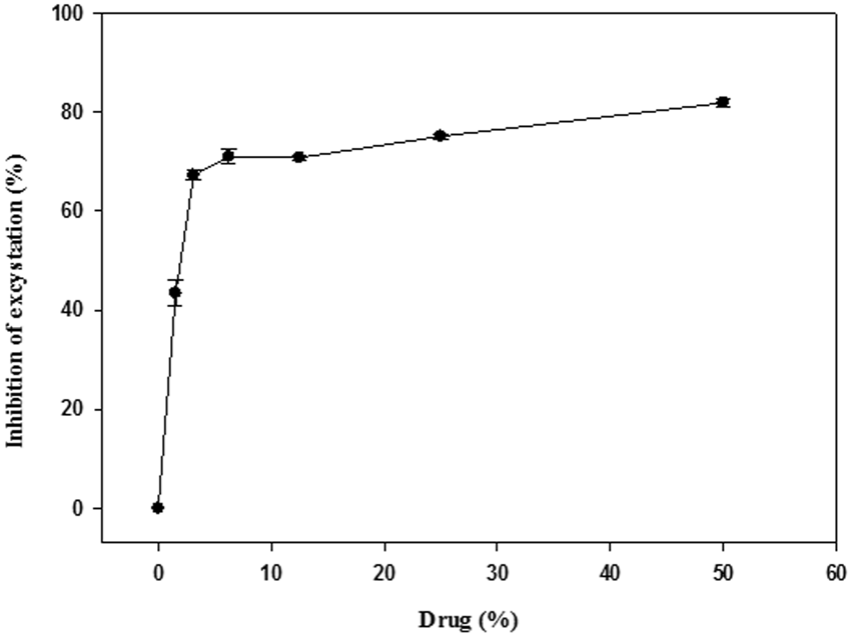
Effect of Eye drop solution ‘Systane Ultra’ on the excystation process of *Acanthamoeba castellanii* Neff.

Systane Ultra was observed to cause a dose-dependent cysticidal affect (Fig. 2). We found that 1.35% of Systane Ultra inhibited 50% excystation from the initial inoculum of cysts.

### Systane Ultra treated cells stained positive in the double-stain assay

**Figure 3:**
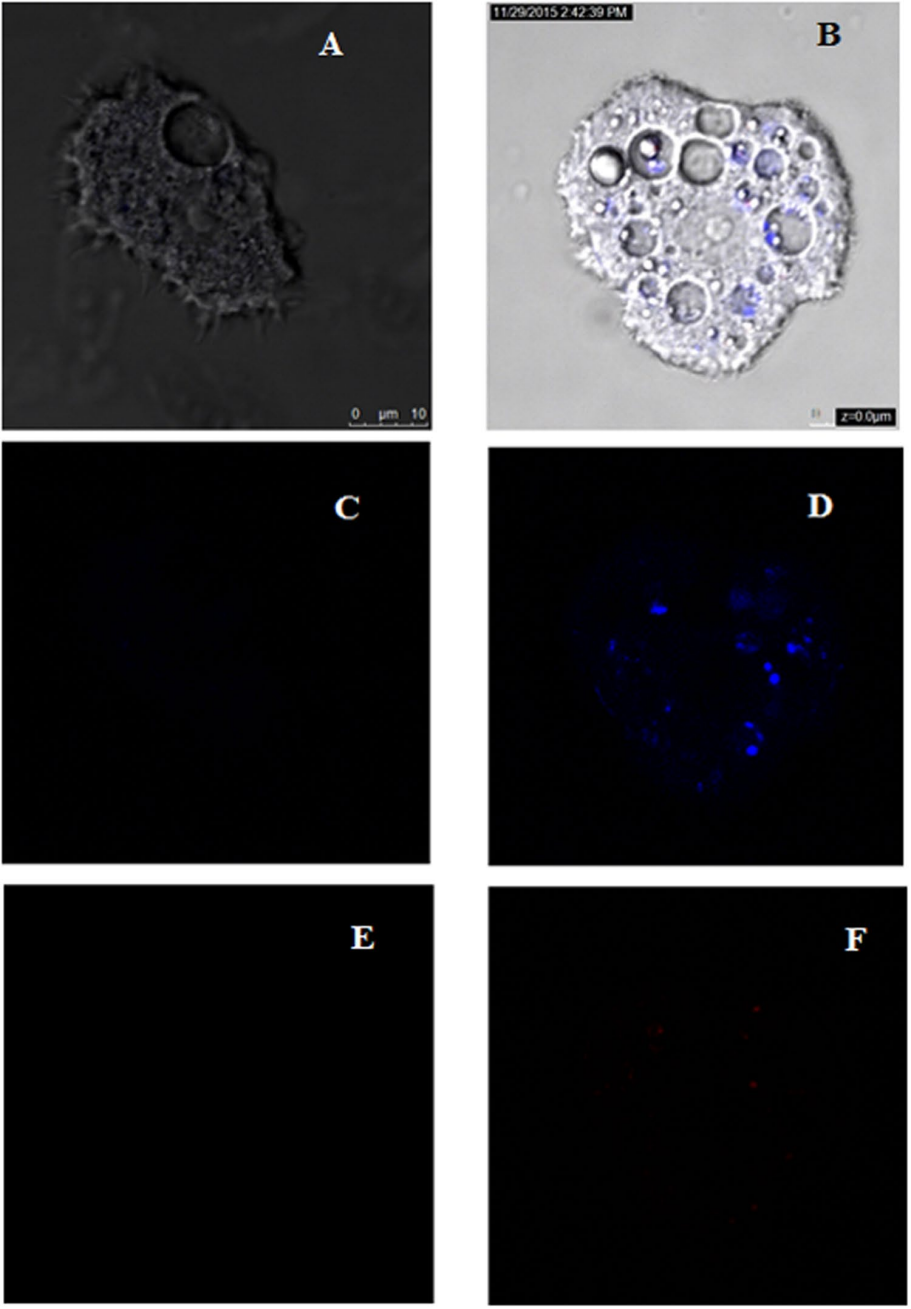
Hoechst staining is different in control cells, where uniformly faint-blue nuclei are observed, and in treated cells, where the nuclei are bright blue. (**A** to **C**) Overlay images: control (24 h) (**A**), Systane Ultra IC_50_ (24 h) (**B**), Systane Ultra IC_90_ (24 h). (**D**–**F**) Hoechst channel: control (24 h) (**D**), Systane Ultra IC_50_ (24 h) (**E**), Systane Ultra IC_90_ (24 h) (**F**). (**G** to **I**) Propidium iodine channel: control (24 h) (**G**), Systane Ultra IC_50_ (24 h) (**H**), Systane Ultra IC_90_ (24 h) (**I**). (Magnification of 64x).

When double staining was performed, the tested drug at a concentration of IC_90_ could induce chromatin condensation proved by the bright-blue nuclei stain as shown by Fig. 3.

### Systane Ultra caused plasma membrane permeability in treated cells

**Figure 4:**
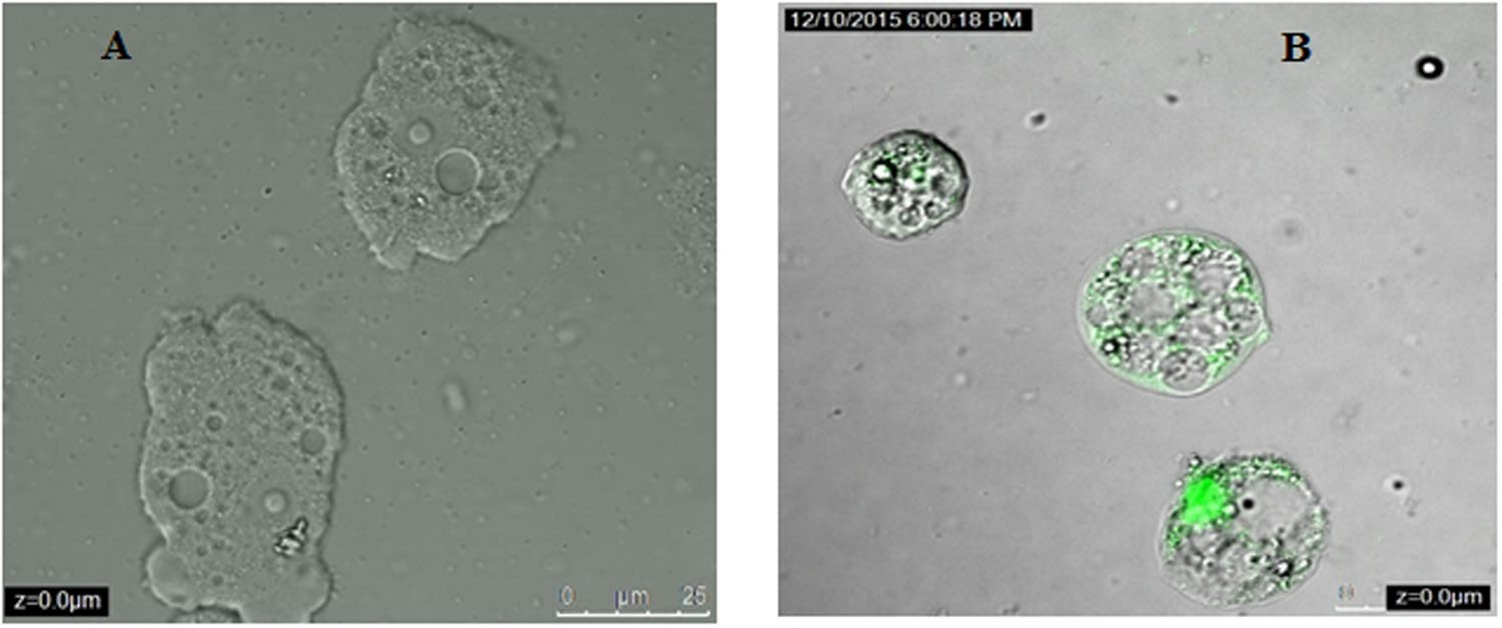
Representative confocal microscopy of *Acanthamoeba castellanii* Neff labeled with SYTOX Green. Parasite were plated as above and incubated for 24 h with IC_90_ of the Systane Ultra (B), Negative control (untreated strains) *Acanthamoeba castellanii*. Neff.

As shown in Fig. 4, amoebae treated with IC_90_ of the tested drug induced cellular membrane damage after 24 hours of treatment. Nevertheless, the cell integrity is still maintained.

### Systane Ultra induced mitochondrial malfunction

**Figure 5:**
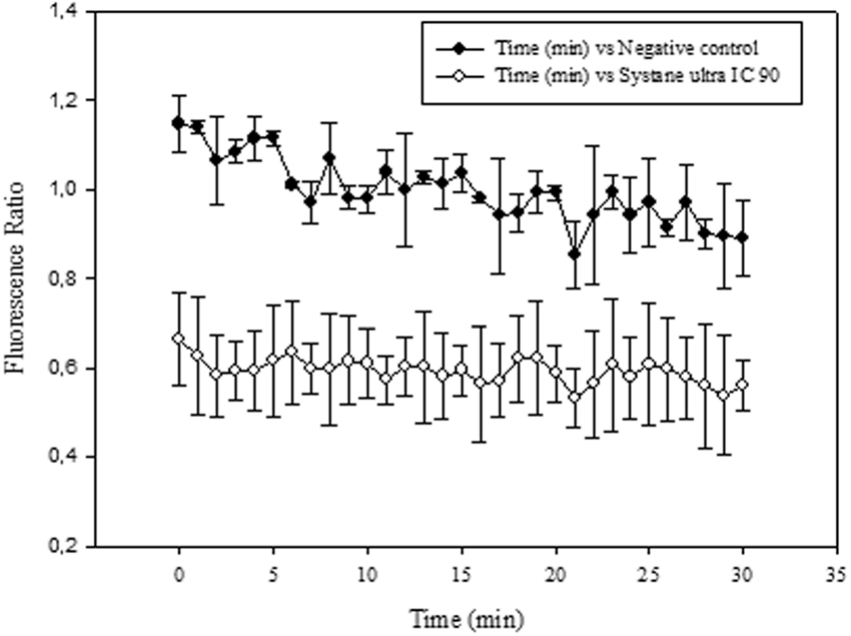
Mitochondrial membrane potential (Δψ_m_) Showing change in the Ratio of fluorescence intensity at 590/530 nm after the 24 hours of treatment with the IC_90_ of Systane Ultra. (Filled diamond shapes vehicle only control, empty diamonds with Systane Ultra).

**Figure 6:**
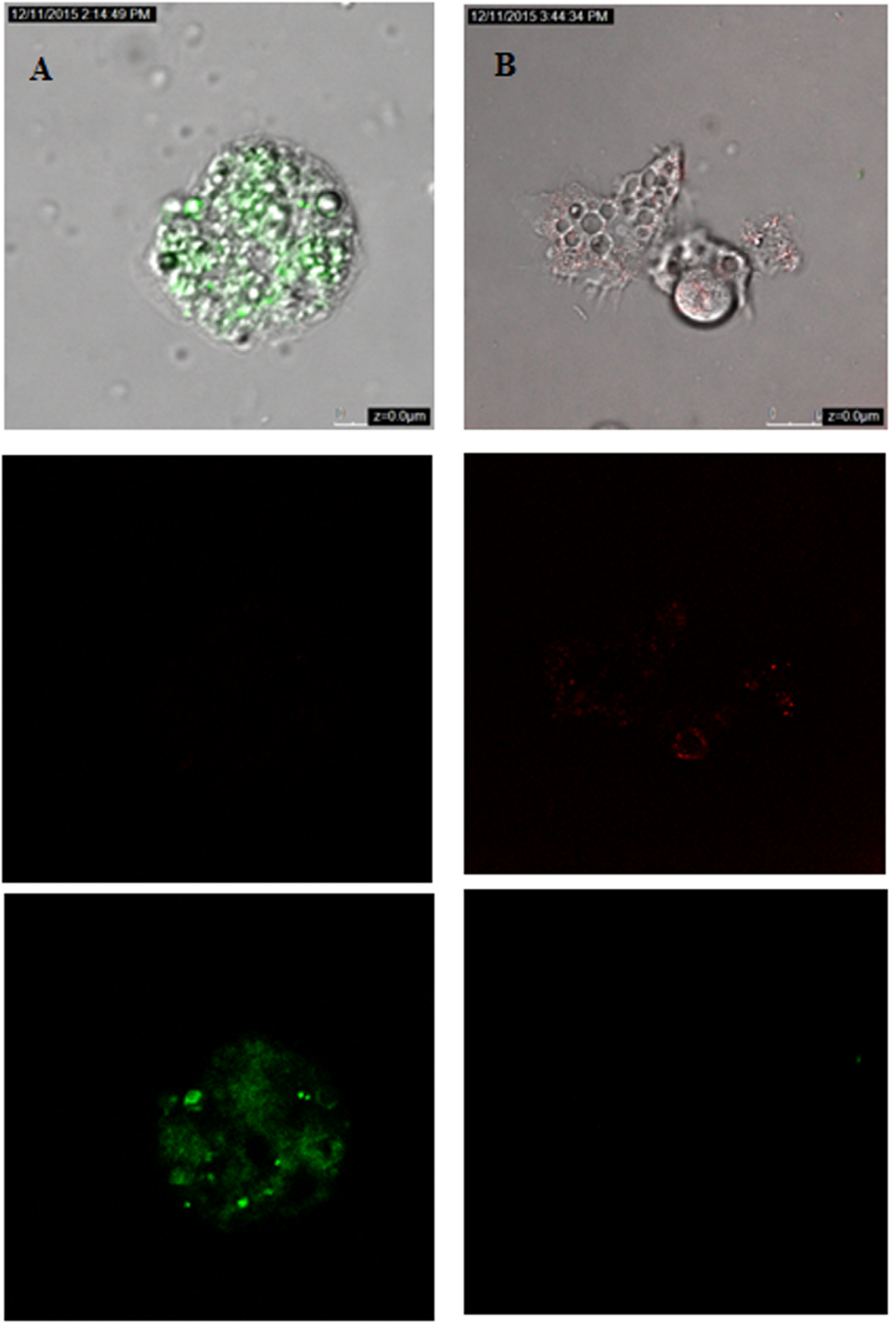
The effect of Systane Ultra on the mitochondrial potential, JC-1 dye accumulates in the mitochondria of healthy cells as aggregates (red fluorescence) (Negative control B); in cells treated with the IC_90_ of Systane Ultra for 24 h, due to collapse of mitochondrial potential, the JC-1 dye remained in the cytoplasm in its monomeric form, green fluorescence. (Images are representative of the population of treated amoebae).

**Figure 7:**
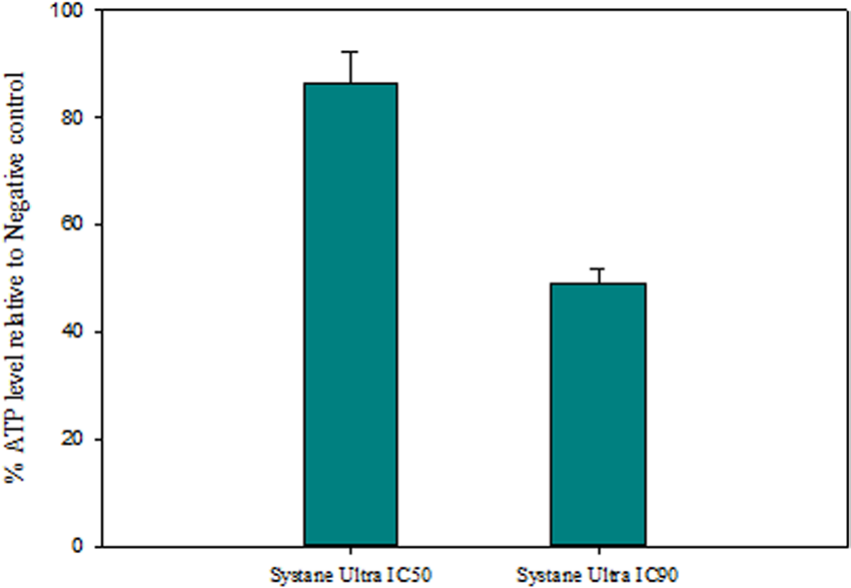
The effect of Systane Ultra on the ATP content, using CellTiter-Glo Luminescent Cell Viability Assay. Results are representing in percentage relative to the negative control. Cells were treated by the IC_50_ and IC_90_ concentration for 24 hours.

As it can be observed in Fig. 5, the curve of the mitochondrial potential fluorescence ratio demonstrated that the treatment with the IC_90_, decreased the membrane potential (Δ*Ψm*) of *A. castellanii* Neff comparing to the negative control. As presented in the Fig. 6, confocal microscopy confirmed the effects of the Systane Ultra on the mitochondrial potential. The mitochondrial damage has been documented by measuring the ATP level generated in 24 h. We found out, that the IC_90_ produced a pronounced decrease in the total ATP level (Fig. 7). In fact, cells treated with this dose generated only the half of ATP level produced in untreated cells.

## Discussion

AK is a vision-threatening ophthalmological illness that may even result in blindness if left untreated. In the early stages of infection, this disease usually manifests with nonspecific symptoms such as eye redness, epithelial defects, photophobia, edema and intense pain. AK is often mis-diagnosed as many of these symptoms are shared with the other eye problems^11,12^. An increase in the number of AK cases is blamed on contact lens use, particularly of soft contact lenses, and their improper use and maintenance^12,13^. The use of contact lenses is also associated with DED^14^, and this is also a predisposing factor in the development of AK^2^. We have described anti-*Acanthamoeba* activities of 9 eye drop solutions using a range of *Acanthamoeba* strains. Among the tested eye drops, Systane Ultra was the most active against all the tested *Acanthamoeba* strains. The fact that the eye drops have an anti-cyst activity has been established by viability and proliferative assay and analyzed by microplate-based fluorescence. The Systane Ultra formulation contains 0.4% Polyethylene Glycol 400 which has been reported^15^ to be effective against various pathogenic bacteria, including *Klebsiella pneumonia*, *Pseudomonas aeruginosa*, *Escherichia coli*, and *Staphylococcus aureus* through damage to bacterial membrane^15,16^. Systane Ultra also contains 0.3% Propylene Glycol, and several reports have described the antimicrobial property of this molecule and its effectiveness as a preservative^17,18^.

The effects of Systane Ultra on *Acanthamoeba* that we describe here are consistent with a Programmed Cell Death (PCD) mechanism. A PCD-like process has been described in *Acanthamoeba* occurring 6 h after infection with *Salmonella typhimurium*^19^. The early stage was inferred from phosphatidylserine externalization and chromatin condensation. Since this initial report we^20,21^ and others^22,23^ have reported a number of PCD inducing agents in *Acanthamoeba*. In these reports, authors have been able to distinguish between early PCD cells, late PCD cells. This process is generally characterized by distinct morphological features that occurs in different stages from the loss of mitochondrial potential, the condensation of nuclear chromatin and exposure of phosphatidylserine (PS) on the cell exterior. At the late of PCD, the membrane starts blebbing and cell dehydration causes changes in cellular shape and size. The structural integrity and most of the functions of the cell membrane remain intact at least in the initial stages of the process^20^. In the present study, Systane Ultra at the IC_90_ was found to induce chromatin condensation observed through the Hoechst fluorescence as showed in the Fig. 3. Some of the brighter staining material is associated with structures within vacuoles and these are likely to be autophagosomes^24^. However, this is still a speculative hypothesis since a complete autophagy evaluation should be performed by analyzing the autophagosome formation among other assays.

To get a better knowledge of the membrane damage caused by Systane Ultra, we measured fluorimetrically the influx of SYTOX Green into the parasites, as its fluorescence is enhanced when bound to intracellular nucleic acids. After 24 hours of treatment, this eye drop solution was able to induce lesions in the plasma-membrane with a size large allowing the entrance of the dye but without cell rupture. The maintenance of cell’s shape was confirmed using confocal microscopy as showed by Fig. 4. It is well known that PCD is linked to the malfunction of the mitochondria. The loss of mitochondrial membrane potential leads to mitochondrial dysfunction and this is regarded as being an important factor in PCD^25^. In the present study, the selected eye drop produced a pronounced decrease in the mitochondrial potential and therefore in the total ATP level. It’s likely that Systane Ultra induces apoptosis in *Acanthamoeba* cells through the mitochondrial pathway.

## Conclusion

Our results suggest that Systane Ultra possess an amoebicidal activity that may be useful for the prevention or even treatment of *Acanthamoeba* keratitis, or form the basis for an optimized solution. We suggest that the Systane Ultra eye drop solution probably induces PCD via the intrinsic pathway. Nevertheless, a limitation of this study is whether these eye drops could be used in the future since they could not be available commercially in ten-year time. Another issue is the need to perform further studies using an *in vivo* model, since the *in vitro* methodology used has its limitations such as the lack of a water related vehicle control and/washing action of tears in the eye once the eye drops are placed on the eye and washed away. Therefore, the need for further studies in the near future using these eye drops and an *in vivo* model.

Nevertheless, the potential use of these eye drops especially Systane Ultra due to its high anti-*Acanthamoeba* effects is clear and presents a promising alternative for AK treatment in the current and near future infection cases.
